# Supplementary material for: Is an Exercise Program for Pediatric Cancer Patients in Palliative Care Feasible and Supportive?—A Case Series
Source: Children (Basel). 2023 Feb 7;10(2):318. doi: 10.3390/children10020318 (PMC9955029; doi:10.3390/children10020318)
Supplement: Supplementary file 1 [file children-10-00318-s001.zip › children-2135022-supplementary.pdf]

CARE case report guidelines

CARE Checklist of information to include when writing a case report

(cc) BY-NC-ND

Topic	Item	Checklist item description	Reported on Line
Title	1	The diagnosis or intervention of primary focus followed by the words "case report"	3
Key Words	2	2 to 5 key words that identify diagnoses or interventions in this case report, including "case report"	22-23
Abstract (no references)	3a	Introduction: What is unique about this case and what does it add to the scientific literature?	8-11
	3b	Main symptoms and/or important clinical findings	11
	3c	The main diagnoses, therapeutic interventions, and outcomes	11-15
	3d	Conclusion—What is the main "take-away" lesson(s) from this case?	19-21
Introduction	4	One or two paragraphs summarizing why this case is unique (**may include references**)	26-43
Patient Information	5a	De-identified patient specific information	51-55
	5b	Primary concerns and symptoms of the patient	51-55
	5c	Medical, family, and psycho-social history including relevant genetic information	52-54,103-112,135-144
	5d	Relevant past interventions with outcomes	104-108, 135-144
Clinical Findings	6	Describe significant physical examination (PE) and important clinical findings	104-108, 135-144
Timeline	7	Historical and current information from this episode of care organized as a timeline	Fig 1
Diagnostic Assessment	8a	Diagnostic testing (such as PE, laboratory testing, imaging, surveys).	63-78
	8b	Diagnostic challenges (such as access to testing, financial, or cultural)	176-178
	8c	Diagnosis (including other diagnoses considered)	51-55
	8d	Prognosis (such as staging in oncology) where applicable	51-55
Therapeutic Intervention	9a	Types of therapeutic intervention (such as pharmacologic, surgical, preventive, self-care)	56-62
	9b	Administration of therapeutic intervention (such as dosage, strength, duration)	56-62
	9c	Changes in therapeutic intervention (with rationale)	56-62
Follow-up and Outcomes	10a	Clinician and patient-assessed outcomes (if available)	87-153
	10b	Important follow-up diagnostic and other test results	Fig 2
	10c	Intervention adherence and tolerability (How was this assessed?)	80-84
	10d	Adverse and unanticipated events	91
Discussion	11a	A scientific discussion of the strengths AND limitations associated with this case report	157-185
	11b	Discussion of the relevant medical literature **with references**	167-170, 180-182
	11c	The scientific rationale for any conclusions (including assessment of possible causes)	167-175
	11d	The primary "take-away" lessons of this case report (without references) in a one paragraph conclusion	187-190
Patient Perspective	12	The patient should share their perspective in one to two paragraphs on the treatment(s) they received	not applicable
Informed Consent	13	Did the patient give informed consent? Please provide if requested	Yes ☒ No ☐
